# Challenging the Traditional Paradigm: Clinical and Functional Outcomes of Early versus Very-Early Subthalamic Deep Brain Stimulation in Parkinson’s Disease

**DOI:** 10.1159/000553293

**Published:** 2026-08-06

**Authors:** Buse Cagla Ari, Ozgun Koksal, Nesrin Helvaci Yilmaz, Mihrimah Sultan Cakmak, Yasemin Ekmis, Eylul Adiguzel, Yavuz Samanci, Erdem Kurt, Gulay Kenangil, Andres M. Lozano, T. Ali Zirh

**Affiliations:** a Istanbul Medipol University, Parkinson’s Disease and Movement Disorders Center (PARMER), Acibadem District Hospital, Istanbul, Turkey; b Istanbul Training and Research Hospital, Istanbul, Turkey; c Department of Neurosurgery, University of Toronto, Toronto, ON, Canada; d Krembil Brain Institute, University Health Network, Toronto, ON, Canada

**Keywords:** Deep brain stimulation, Movement disorder surgery, Parkinson’s disease, Subthalamic nucleus, Early deep brain stimualtion, Movement disorders

## Abstract

**Introduction:**

Subthalamic nucleus deep brain stimulation (STN-DBS) is an established treatment for advanced Parkinson’s disease (PD). While traditionally reserved for later stages, growing evidence suggests earlier intervention may offer benefits. This study compares outcomes of early versus very-early DBS in a single-center cohort.

**Methods:**

This retrospective study included 65 PD patients undergoing STN-DBS (2015–2025), grouped as very-early (<5 years disease duration, *n* = 32) or early (≥5 years, *n* = 33). Groups were comparable in age, sex, and disease severity. Results included Hoehn and Yahr (H&Y), UPDRS-III, MMSE, and levodopa equivalent daily dose (LEDD) assessed preoperatively, early postoperatively, and at 3 years.

**Results:**

Mean age was 62.45 ± 8.79 years; 69.2% were male. Both groups showed significant postoperative improvement in H&Y (*p* < 0.001), with partial decline at 3 years, though still better than baseline. No significant group-time interaction was observed for H&Y or UPDRS-III, indicating similar long-term motor outcomes. The group-by-time interaction for LEDD was significant (*p* = 0.002), reflecting different medication trajectories: both groups converged to similar postoperative LEDD levels despite the early DBS group starting from a substantially higher baseline (1,010 ± 514 vs 676 ± 394 mg/day; *p* = 0.005). Multivariable regression showed that baseline preoperative LEDD was the dominant predictor of postoperative medication reduction; after adjustment, surgical timing was no longer significantly associated with LEDD reduction (*p* = 0.622). Very-early DBS patients had lower early postoperative H&Y scores (*p* = 0.022). Six patients were reclassified as PD plus during follow-up; sensitivity analyses yielded consistent results.

**Conclusion:**

Both very-early and early STN-DBS provide significant short-term motor benefits, though some decline occurs over time. Very-early DBS was associated with greater postoperative medication reduction and more favorable early functional outcomes, while long-term motor outcomes remained comparable between groups. However, differences in baseline medication burden may have contributed to the observed LEDD reduction patterns.

## Introduction

Deep brain stimulation (DBS) targeting the subthalamic nucleus is a well-established surgical treatment for Parkinson’s disease (PD). Its superiority over optimal medical therapy has been demonstrated in numerous randomized controlled trials [1, 2]. However, for many years, DBS was reserved for patients with advanced disease, particularly those with persistent motor fluctuations and dyskinesias despite careful and prolonged pharmacological optimization. The prevailing view was that the risks of surgery were justified only when medical treatment could no longer provide satisfactory symptom control [3]. This view has been fundamentally challenged by the EARLYSTIM randomized controlled trial. The study showed that combining STN-DBS with best medical treatment resulted in significantly greater improvement in quality of life and a reduction in motor complications compared to medical treatment alone, even in patients with relatively short-term motor fluctuations and an average disease duration of approximately 7.5 years at enrollment [4]. In light of these findings, the US Food and Drug Administration (FDA) has expanded the indication for DBS to include patients with at least 4 years of motor complications [5]. Neurophysiologically, chronic pulsatile dopaminergic stimulation is thought to lead to maladaptive reorganization in basal ganglia circuits. Performing surgical intervention before these changes are finalized may help alleviate or delay the long-term effects of the disease’s clinical manifestations [6].

There is currently no universally accepted definition of “early DBS” in the PD literature, and the terminology remains heterogeneous across studies. While some authors define early intervention as surgery performed prior to the development of advanced motor complications, others have used broader thresholds ranging from approximately 4 to 8 years after diagnosis or symptom onset [7, 8]. In the EARLYSTIM trial, for example, the mean disease duration at the time of surgery was approximately 7.5 years, and recent meta-analytic data similarly classified DBS performed within less than 8 years of disease duration as “early” intervention [9]. This inevitably limits the extent to which these findings can be applied to larger, unselected clinical populations [4, 10]. Long-term data are also insufficient, particularly regarding functional outcomes over time, changes in levodopa-equivalent daily dose (LEDD), and adverse-event profiles associated with the timing of surgery [11]. Furthermore, it remains unclear which patient subgroups benefit most from early surgical intervention. This question has not been systematically investigated in single-center, real-world registries, where referral patterns, patient characteristics, and follow-up practices often differ significantly from controlled trial settings [12, 13]. Consequently, translating trial-based evidence into daily clinical decision-making remains challenging.

This study investigates PD patients treated at our referral center and compares those who underwent DBS within 5 years of diagnosis with those who underwent surgery 5 years or more after diagnosis. The present study, therefore, primarily investigates outcomes associated with very-early DBS implantation compared with early intervention within a real-world clinical cohort. We evaluated motor outcomes related to the timing of surgical intervention, health-related quality of life, changes in LEDD, and the incidence of adverse events.

## Methods

This retrospective cohort study was conducted at the Istanbul Medipol University Parkinson’s Disease and Movement Disorders Center (PARMER), a referral center specializing in movement disorders. We reviewed the medical records of patients who underwent DBS surgery between January 2015 and December 2025. All procedures were performed by the same functional neurosurgery team using standard stereotactic techniques, and postoperative programming was managed by the same movement disorders neurology team throughout the study period, ensuring consistency in both the procedure and follow-up. Patients who fulfilled Movement Disorders Society (MDS) clinical diagnostic criteria [14] for PD at the time of surgery were included in the primary analyses, even if subsequent longitudinal follow-up led to diagnostic reclassification to atypical parkinsonian syndromes. The patient cohort included individuals who underwent DBS surgery at our institution, were 18 years or older at the time of surgery, and had complete preoperative documentation with at least 3 years of postoperative follow-up.

**Fig. 1.**
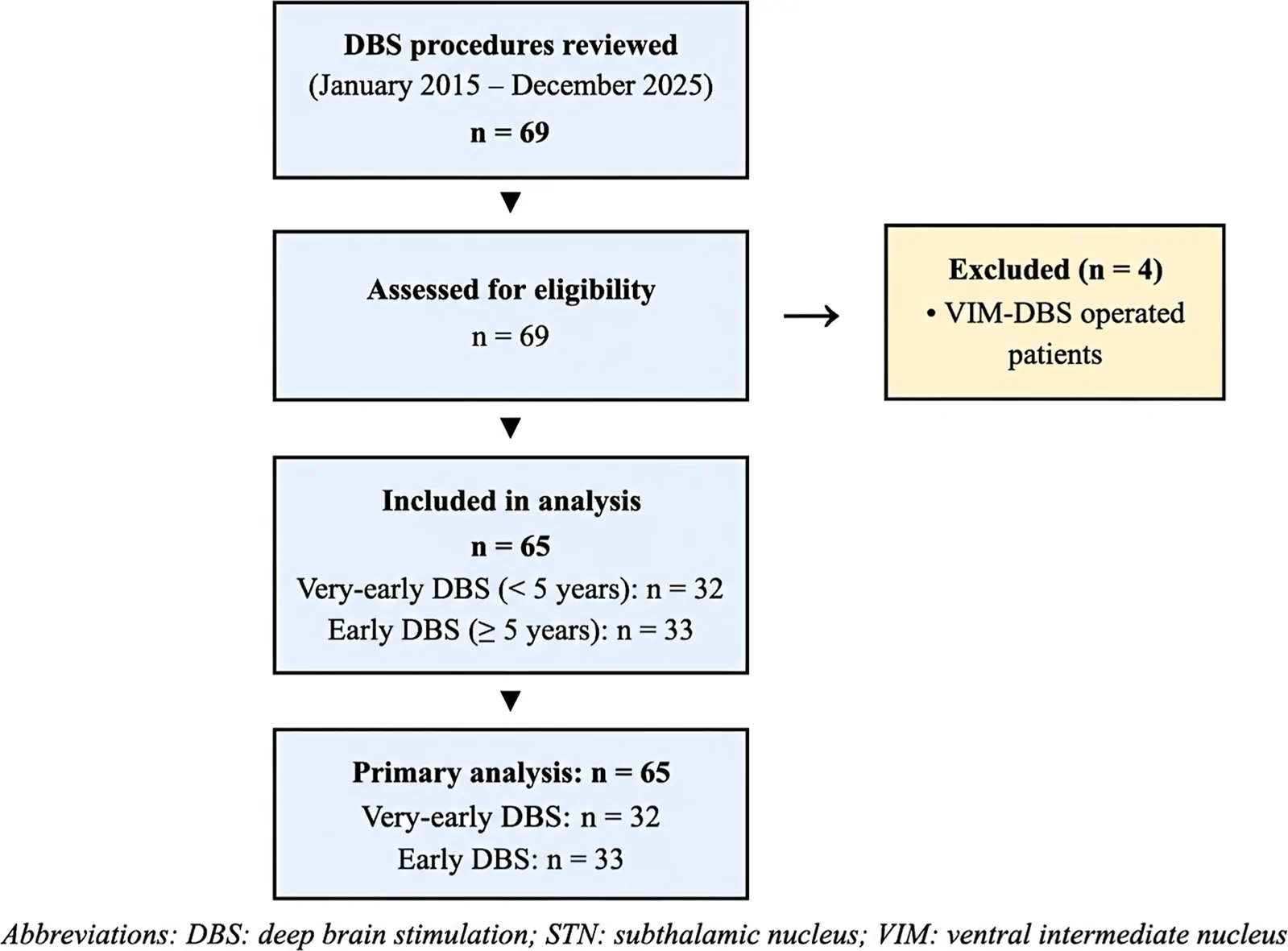
Flow diagram of the patient selection process. A total of 152 patients who underwent subthalamic nucleus deep brain stimulation (STN-DBS) between 2015 and 2025 were initially screened. Following the application of exclusion criteria – including cognitive impairment (*n* = 28), secondary dystonia (*n* = 12), and incomplete follow-up data (*n* = 47) – 65 patients were included in the final analysis. The study population was categorized into two groups based on the time from the diagnosis to surgery: very-early STN-DBS (<5 years, *n* = 32) and early STN-DBS (≥5 years, *n* = 33).

Exclusion criteria included patients with postural instability and gait difficulty type PD, initial atypical or secondary parkinsonism, prior intracranial surgery, cognitive impairment (Mini-Mental State Examination [MMSE], Turkish version, score <24) [15], active major psychiatric illness incompatible with informed consent, or incomplete follow-up data. To reduce selection bias, all consecutive eligible patients identified through the institution’s DBS registry were included in the study. Patients were divided into 2 groups for analysis based on the time from diagnosis to surgery: the “*very-early DBS*” group (surgery within 60 months of symptom onset) and the “*early DBS*” group (surgery 60 months or more after symptom onset) (shown in Fig. 1).

Age at surgery, gender, age at diagnosis, clinical subtype (tremor-dominant [TD], akinetic-rigid [AR], or postural instability and gait difficulty), disease duration, follow-up duration, and time from diagnosis to DBS were recorded. We also documented LEDD values preoperatively, postoperatively, and 3 years after surgery. Baseline variables at the preoperative examination included Unified Parkinson’s Disease Rating Scale part III (UPDRS-III) scores [16], Hoehn and Yahr (H&Y) stage [17], LEDD, and MMSE scores. Preoperative motor assessments were performed using standardized medication-state examinations as part of routine DBS candidacy evaluation. UPDRS-III scores included off-medication assessments obtained after appropriate dopaminergic medication withdrawal according to institutional DBS protocols. Postoperative longitudinal motor evaluations were primarily based on routine off-medication DBS follow-up assessments whenever available. However, due to the retrospective real-world nature of the study, some long-term follow-up visits were conducted under routine clinical conditions rather than fully standardized research protocols, limiting complete separation of stimulation and medication effects across all assessments. Cases where the diagnosis was subsequently revised (e.g., progressive supranuclear palsy [PSP], multiple system atrophy [MSA], or corticobasal syndrome [CBS]) were documented as well. Clinical assessments were conducted prospectively at predefined visits (preoperative, postoperative, and 3 years post-surgery). LEDD was calculated using standardized conversion formulas [18]. Data extraction was performed by 2 independent researchers (B.C.A., G.K.), and to reduce information bias, 20% of records were randomly verified.

The normality of continuous variables was assessed using graphical methods and the Shapiro-Wilk test. Descriptive statistics were shown as mean ± standard deviation or median (minimum-maximum), depending on data distribution. Categorical variables were compared between the early and very-early DBS groups using contingency tables, with results expressed as counts (*n*), percentages (%), and chi-square (χ^2^) statistics. Clinical variables were compared between groups using the Mann-Whitney U test. Changes between 2 timepoints (postoperative and year 3 postoperative) were evaluated with the Wilcoxon signed rank test. For comparisons across 3 timepoints (preoperative, postoperative, and year 3 postoperative), the Friedman test for related samples was used. Differences in clinical scores over time between the early and very-early DBS groups were examined using two-way mixed ANOVA. When ANOVA assumptions were not met, nonparametric longitudinal analysis was performed with the nparLD package in *R* (F1-LD-F1 design). Correlations were assessed using Spearman’s nonparametric correlation coefficient. To evaluate the robustness of the primary findings, a prespecified sensitivity analysis was performed excluding patients who were reclassified as PD-plus syndromes during follow-up. Additionally, multivariable linear regression models were constructed to assess whether between-group differences persisted after adjustment for baseline confounders. LEDD reduction models were adjusted for age at surgery and baseline preoperative LEDD. UPDRS-III improvement models were adjusted for age and baseline preoperative UPDRS-III scores. Postoperative H&Y stage was adjusted for age and baseline H&Y stage. Disease duration was not included as a covariate due to strong collinearity with the group variable (*r* = −0.72). Residual diagnostics included heteroscedasticity-consistent (HC3) standard errors and nonparametric bootstrap resampling (B = 2000). The F1-LD-F1 design was implemented in *R* with RStudio. All other statistical analyses used IBM SPSS Statistics version 26.0 (IBM Corp., Armonk, NY, USA) and Microsoft Excel 2024. A *p* value <0.05 was considered statistically significant.

## Results

### Participant Characteristics

A total of 65 PD patients who underwent STN-DBS were included in the study: 32 (49.2%) were in the very-early DBS group, and 33 (50.8%) were in the early DBS group. The mean age of the cohort was 62.45 ± 8.79 years (median 65.0, range 36–79), and 69.2% were male. The mean follow-up duration was 3.05 ± 1.65 years (median 3.0 years), and there was no significant difference between the groups (*p* = 0.814). The distribution of disease subtypes was similar between groups: 43.1% of the total cohort had TD subtype PD, and 49.2% had AR subtype PD (*p* = 0.797). There were no differences between the 2 groups in terms of age at the time of surgery (60.71 ± 11.76 vs. 64.12 ± 3.86 years; *p* = 0.340), gender (*p* = 0.934), or postoperative disease duration (*p* = 0.178). In accordance with the group definitions, the time from diagnosis to DBS surgery (5.16 ± 1.79 years vs. 10.12 ± 3.69 years; *p* < 0.001) and preoperative LEDD (675.75 ± 393.54 mg/day vs. 1,010.03 ± 514.38 mg/day; *p* = 0.005) were significantly higher in the early DBS group. The age at initial diagnosis was higher in the very-early DBS group (55.56 ± 11.33 vs. 54.00 ± 4.37 years; *p* = 0.039). Demographic and clinical characteristics are summarized in Table 1.

**Table 1.** Baseline demographic and clinical characteristics of very-early and early DBS groups

Variables	All patients (*n* = 65)	Very-early DBS <5 years (*n* = 32)	Early DBS ≥5 years (*n* = 33)	z; χ^2^	*p* value
Age, mean ± SD, years	62.45±8.79	60.71±11.76	64.12±3.86	*z* = 0.954	0.340
Median (range)	65.0 (36–79)	63.5 (36–79)	65.0 (49–71)		
Female	20 (30.8)	10 (31.3)	10 (30.3)	χ^2^ = 0.007	0.934
Male	45 (69.2)	22 (68.7)	23 (69.7)		
Follow-up, mean ± SD, years	3.05±1.65	3.03±1.45	3.06±1.85	*z* = 0.235	0.814
TD	28 (43.1)	13 (40.6)	15 (45.5)	χ^2^ = 0.454	0.797
AR	32 (49.2)	17 (53.1)	15 (45.5)		
Mixed	5 (7.7)	2 (6.3)	3 (9.1)		
Symptom onset to DBS, mean ± SD, years	5.78±3.60	3.06±0.88	8.42±3.26	*z* = 6.983	**<0.001ʳ**
Diagnosis to DBS, mean ± SD, years	5.23±3.71	2.50±1.19	7.88±3.39	*z* = 6.764	**<0.001ʳ**
Age at first diagnosis, mean ± SD, years	54.77±8.51	55.56±11.33	54.00±4.37	*z* = 2.061	**0.039ʳ**
Preoperative LEDD, mean ± SD, mg/day	845.46±485.46	675.75±393.54	1,010.03±514.38	*z* = 2.809	**0.005ʳ**
MMSE, mean ± SD	27.89±1.62	28.00±1.46	27.79±1.78	*z* = 0.156	0.876
Preoperative H&Y stage, mean ± SD	2.32±0.61	2.25±0.67	2.39±0.56	*z* = 1.151	0.250
Preoperative UPDRS-III, mean ± SD	31.58±14.962	32.03±14.54	31.15±14.90	*z* = 0.335	0.738
Postoperative H&Y stage, mean ± SD	2.06±0.43	1.94±0.43	2.19±0.39	*z* = 0.293	0.022*
Postoperative UPDRS-III, mean ± SD	13.86±8.74	14.56±8.94	13.18±8.62	*z* = 0.919	0.361
3-Year H&Y stage, mean ± SD	2.84±0.70	2.90±0.66	2.79±0.74	*z* = 0.666	0.506
3-Year UPDRS-III, mean ± SD	19.54±11.24	19.50±9.96	19.58±12.52	*z* = 0.467	0.641
PD-plus reclassification, *n* (%)	6 (9.2)	5 (15.6)	1 (3.0)	–	0.091^n^

UPDRS-III values primarily reflect off-medication assessments obtained during routine DBS follow-up evaluations.

DBS, deep brain stimulation; H&Y, Hoehn and Yahr; LEDD, levodopa equivalent daily dose; MMSE, Mini-Mental State Examination; STN, subthalamic nucleus; UPDRS, Unified Parkinson’s Disease Rating Scale; VIM, ventral intermediate nucleus.

*p < 0.05.

ʳWelch’s *t* test.

^n^Fisher’s exact test.

^χ2^chi-square test.

Bold values mean statistically significant.

### Longitudinal Changes over Time (Preoperative to 3-Year Follow-Up)

In both groups, a statistically significant improvement was observed in all clinical parameters during the early postoperative period compared with baseline values; however, some deterioration occurred over the 3-year follow-up period, consistent with disease progression. In both groups, scores remained higher during the 3-year follow-up than preoperative levels. Concerning the H&Y stage, intra-group improvements were significant in both the very-early DBS group (χ^2^ = 33.811; *p* < 0.001) and the early DBS group (χ^2^ = 23.094; *p* < 0.001). Nonetheless, the group × time interaction was not statistically significant (*p* = 0.087). For UPDRS-III, both groups demonstrated significant within-group improvements (very-early: χ^2^ = 45.016, *p* < 0.001; early: χ^2^ = 55.569, *p* < 0.001), but the group × time interaction was not significant (*p* = 0.788). Total LEDD showed a statistically significant decrease within the group compared to baseline in both the very-early DBS group (χ^2^ = 8.968, *p* = 0.011) and the early DBS group (χ^2^ = 40.828, *p* < 0.001). Importantly, the group × time interaction for LEDD was statistically significant (*p* = 0.002), indicating that the pattern of change in medication requirements over time differed between groups. Both groups reached similar postoperative LEDD levels despite different baseline values (very-early DBS: from 675.75 ± 393.54 to 370.72 ± 287.04 mg/day; early DBS: from 1,010.03 ± 514.38 to 372.08 ± 222.56 mg/day). Since the early-DBS group started with a higher baseline LEDD, it demonstrated a greater absolute reduction over time. In multivariable regression analyses adjusted for age and baseline preoperative LEDD, baseline LEDD emerged as the main predictor of postoperative medication reduction (*p* < 0.001). After adjustment, surgical timing was no longer significantly associated with postoperative LEDD reduction (B = −34.56; 95% CI: −173.92 to 104.81; *p* = 0.622), while only a borderline association remained for LEDD reduction at 3-year follow-up (B = −159.61; 95% CI: −324.06 to 4.84; *p* = 0.057). These findings are presented in Table 2.

**Table 2.** Longitudinal clinical outcomes by group across three timepoints

Parameters	Timepoint	Very-early DBS (*n* = 32), mean ± SD	Early DBS (*n* = 33), mean ± SD	*p* (within group)	*p* (GxT interaction)
H&Y stage	Preop	2.25±0.67	2.39±0.56	**Both <0.001ʳ**	0.087
	Postop	1.94±0.43	2.19±0.39		
	3 years	2.90±0.66	2.79±0.74		
UPDRS-III	Preop	32.03±14.54	31.15±14.90	**Both <0.001ʳ**	0.788
	Postop	14.56±8.94	13.18±8.62		
	3 years	19.50±9.96	19.58±12.52		
Total LEDD, mg/day	Preop	675.75±393.54	1,010.03±514.38	Early: *p* = 0.011; late: ***p* <0.001ʳ**	0.002*
	Postop	370.72±287.04	372.08±222.56		
	3 years	470.04±320.61	416.61±337.50		

GxT, group-by-time interaction; H&Y, Hoehn and Yahr; LEDD, levodopa equivalent daily dose; UPDRS, Unified Parkinson’s Disease Rating Scale.

*p < 0.05 for GxT interaction.

ʳFriedman test.

Bold values mean statistically significant.

Among the 6 patients who were subsequently reclassified as PD-plus syndromes, 5 belonged to the very-early DBS group and 1 to the early DBS group. Final diagnoses included PSP, MSA, and CBS. Although several of these patients initially demonstrated partial postoperative improvement in tremor or motor fluctuations, long-term follow-up revealed comparatively less favorable progression trajectories, particularly regarding axial symptoms, postural instability, gait dysfunction, and overall functional decline.

### Disease Progression from Early Postoperative Period to 3-Year Follow-Up

From the early postoperative assessment through the 3-year follow-up, both groups showed a significant worsening in H&Y stage (very-early DBS: *z* = 4.460, *p* < 0.001; early DBS: *z* = 4.243, *p* < 0.001) and in UPDRS-III scores (very early: *z* = 4.113, *p* < 0.001; early: *z* = 4.356, *p* < 0.001). The group × time interaction for this interval was significant for the H&Y stage (*p* = 0.014), indicating that the 2 groups began this progression process from different functional starting points: the very-early DBS group had a significantly lower postoperative baseline H&Y score (1.94 ± 0.43 vs. 2.19 ± 0.39) and was therefore in a more favorable functional position throughout this progression phase. No significant group × time interaction was detected for UPDRS-III during this interval (*p* = 0.434), indicating that the rate of deterioration in motor scores was similar between groups.

### Subgroup Analyses by Disease Subtype

Among TD subtype PD patients (very-early DBS *n* = 13; early DBS *n* = 15), both groups showed significant worsening in the H&Y stage (very early: *p* = 0.002; early: *p* = 0.002) and UPDRS-III (very early: *p* = 0.003; early: *p* = 0.006). No significant group × time interaction was observed for any parameter (H&Y: *p* = 0.110; UPDRS-III: *p* = 0.334), indicating that DBS timing does not differently affect motor progression in this subtype. A similar pattern was observed among patients with AR subtype PD (very-early DBS, *n* = 17; early DBS, *n* = 15). Both groups showed significant postoperative improvement in the H&Y stage (very early: *p* = 0.002; early: *p* = 0.014) and UPDRS-III (very early: *p* = 0.004; early: *p* = 0.001). Group × time interactions were not significant for any parameter (H&Y: *p* = 0.053; UPDRS-III: *p* = 0.973). This aligns with the finding that, in the TD subgroup, the timing of surgery does not affect the rate of motor progression regardless of the disease phenotype. These results are shown in Table 3.

**Table 3.** Postoperative and 3-year clinical outcomes stratified by disease subtype

Parameters	Timepoint	Very-early DBS, mean ± SD	Early DBS, mean ± SD	*p* (within group)	*p* (GxT)
TD subtype (very-early DBS: *n* = 13; early DBS: *n* = 15)					
H&Y stage	Postop	2.08±0.8	2.27±0.46	**Both *p* <0.05** ^**a**^	0.110
	3 years	3.08±0.76	2.93±0.70		
UPDRS Part III	Postop	15.31±10.93	13.73±8.69	**Both *p* <0.05** ^**a**^	0.334
	3 years	19.69±9.94	21.47±12.09		
AR subtype (very-early DBS: *n* = 17; early DBS: *n* = 15)					
H&Y stage	Postop	1.88±0.48	2.14±0.36	**Both *p* <0.05** ^**a**^	0.053
	3 years	2.80±0.56	2.67±0.82		
UPDRS Part III	Postop	14.71±7.59	14.27±8.83	**Both *p* <0.05** ^**a**^	0.973
	3 years	19.76±10.64	19.40±13.56		

GxT, group-by-time interaction; H&Y, Hoehn and Yahr; UPDRS, Unified Parkinson’s Disease Rating Scale.

^a^Friedman test (within-group comparison across timepoints).

Bold values mean statistically significant.

### Sensitivity Analysis excluding PD-Plus Reclassified Cases

To evaluate whether diagnostic reclassification influenced the primary findings, a sensitivity analysis was performed excluding the 6 patients who were subsequently reclassified as PD-plus syndromes (very-early DBS: *n* = 5; early DBS: *n* = 1), yielding a reduced cohort of 59 patients (very-early DBS: *n* = 27; early DBS: *n* = 32). The overall pattern of results remained consistent with the primary analysis. The greater absolute LEDD reduction in the early DBS group persisted at both the postoperative (*p* = 0.005) and 3-year (*p* = 0.002) timepoints, and between-group UPDRS-III outcomes remained comparable across all assessments. The postoperative H&Y stage advantage in the very-early DBS group was preserved (*p* = 0.025). No reversal in the direction of any primary outcome was observed (Table 4).

**Table 4.** Sensitivity analysis excluding patients re-classified as PD-plus syndromes (*n* = 59)

​	Very-early DBS (*n* = 27), mean±SD	Early DBS (*n* = 32), mean±SD	*p*	Primary analysis *p*
Preoperative LEDD, mg/day	656.65±406.38	1,029.09±510.63	0.002*	**0.005**
Postoperative LEDD, mg/day	358.56±294.71	366.02±223.34	0.784	*NS*
3-Year LEDD, mg/day	395.27±283.13	390.48±309.91	0.790	*NS*
LEDD reduction, postoperative, mg	298.09±404.52	663.07±566.30	0.005*	0.010*
LEDD reduction, 3 years, mg	261.38±413.96	638.61±464.10	0.002*	0.003*
Preoperative UPDRS-III	31.07±14.68	30.00±13.57	0.849	0.738
Postoperative UPDRS-III	12.78±6.37	12.47±7.71	0.593	0.361
3-Year UPDRS-III	17.89±8.96	18.69±11.61	0.915	0.641
Preoperative H&Y stage	2.22±0.70	2.38±0.55	0.229	0.250
Postoperative H&Y stage	1.93±0.38	2.16±0.37	0.025	0.022
3-Year H&Y stage	2.81±0.57	2.75±0.72	0.626	0.506

Mann-Whitney U test and independent-samples *t* test used as appropriate based on distribution. Primary analysis *p* refers to the corresponding between-group comparison in the full cohort (*N* = 65).

NS, not significant in primary analysis; DBS, deep brain stimulation; H&Y, Hoehn and Yahr; LEDD, levodopa equivalent daily dose; UPDRS, Unified Parkinson’s Disease Rating Scale; VIM, ventral intermediate nucleus.

*p < 0.05.

Bold values mean statistically significant.

### Multivariable Regression Adjusting for Baseline Confounders

Given that baseline preoperative LEDD was significantly higher in the early-DBS group, multivariable linear regression was used to evaluate whether the observed between-group differences in LEDD reduction persisted after adjustment for potential confounders. Disease duration was excluded as a covariate because of its strong collinearity with the group variable (*r* = −0.72, *p* < 0.001). Each LEDD outcome was adjusted for age at surgery and baseline preoperative LEDD; each UPDRS-III outcome was adjusted for age and baseline preoperative UPDRS-III; and postoperative H&Y stage was adjusted for age and baseline H&Y stage. After adjustment, surgical timing group was not associated with postoperative LEDD reduction (B = −34.56; 95% CI: −173.92 to 104.81; *p* = 0.622). For 3-year LEDD reduction, the adjusted group effect was borderline (B = −159.61; 95% CI: −324.06 to 4.84; *p* = 0.057). Baseline preoperative LEDD was the dominant predictor at both timepoints (*p* < 0.001), accounting for the majority of explained variance. UPDRS-III improvement did not differ between groups at either timepoint after adjustment (postoperative: *p* = 0.421; 3 years: *p* = 0.956). The postoperative H&Y stage advantage for the very-early DBS group showed a trend toward significance after adjustment (B = −0.180; 95% CI: −0.364 to 0.004; *p* = 0.055). Heteroscedasticity-consistent standard errors and bootstrap resampling (B = 2000) yielded concordant results across all models (Table 5).

**Table 5.** Multivariable linear regression: adjusted association of surgical timing group with postoperative outcomes (*n* = 65)

Outcome/predictor	B	95% CI	*p* value	HC3 p	*R* ^2^
LEDD reduction, postoperative, mg					
Very-early DBS	−34.56	−173.92, 104.81	0.622	0.651	0.762
Age at surgery, years	−1.60	−9.09, 5.89	0.671	0.658	
Baseline LEDD, mg	0.91	0.77, 1.05	<0.001*	<0.001	
LEDD reduction, 3 years, mg					
Very-early DBS	−159.61	−324.06, 4.84	0.057	0.050	0.649
Age at surgery, years	−3.95	−12.79, 4.89	0.375	0.231	
Baseline LEDD, mg	0.75	0.59, 0.92	<0.001*	<0.001	
UPDRS-III improvement, postoperative					
Very-early DBS	−1.21	−4.18, 1.77	0.421	0.386	0.666
Age at surgery, years	−0.07	−0.24, 0.11	0.451	0.515	
Baseline UPDRS-III	0.55	0.44, 0.65	<0.001*	<0.001	
UPDRS-III improvement, 3 years					
Very-early DBS	−0.12	−4.61, 4.36	0.956	0.958	0.448
Age at surgery, years	−0.19	−0.45, 0.07	0.156	0.156	
Baseline UPDRS-III	0.50	0.34, 0.65	<0.001*	<0.001	
Postoperative H&Y stage					
Very-early DBS	−0.180	−0.364, 0.004	0.055	0.054	0.350
Age at surgery, years	0.007	−0.004, 0.018	0.194	0.314	
Baseline H&Y stage	0.375	0.223, 0.527	<0.001*	0.002	

LEDD models adjusted for age at surgery and baseline preoperative LEDD. UPDRS-III models adjusted for age at surgery and baseline preoperative UPDRS-III. H&Y model adjusted for age at surgery and baseline H&Y stage. Disease duration was excluded due to collinearity with group (*r* = −0.72). Bootstrap validation (B = 2,000) confirmed that confidence intervals were concordant across all models.

DBS, deep brain stimulation; H&Y: Hoehn and Yahr; LEDD, levodopa equivalent daily dose; UPDRS, Unified Parkinson’s Disease Rating Scale; VIM, ventral intermediate nucleus; B, unstandardized regression coefficient; HC3, heteroscedasticity-consistent robust standard errors.

*p < 0.05.

Bold values mean statistically significant.

### Correlation Analyses

In the very-early DBS group, age did not show a significant correlation with any clinical parameter at any timepoint (all *p* > 0.05). In the early DBS group, age showed a weak positive correlation with preoperative LEDD (*r* = 0.415, *p* = 0.016), suggesting a possible association between older age at surgery in patients with longer disease duration, higher baseline medication requirements, and less favorable 3-year functional outcomes. Regarding symptom duration, the very-early DBS group showed a positive correlation among postoperative 3rd year H&Y stage (*r* = 0.437, *p* = 0.016). The early DBS group showed no positive correlations. These findings indicate that in patients who undergo surgery at later stages of the disease, a longer preoperative symptom duration is systematically associated with worse motor outcomes at multiple postoperative timepoints. Correlations are summarized in Table 6.

**Table 6.** Spearman correlation coefficients between age, symptom duration, and postoperative clinical outcomes

Clinical variables	Age		Symptom duration	
	very-early DBS	early DBS	very-early DBS	early DBS
Postop 3-year H&Y stage	*r* = 0.124	*r* = 0.326	*r* = 0.437*	*r* = 0.131
	*p* = 0.516	*p* = 0.064	*p* = 0.016	*p* = 0.468
Preoperative LEDD	*r* = −0.200	*r* = 0.415*	*r* = 0.114	*r* = 0.247
	*p* = 0.272	*p* = 0.016	*p* = 0.533	*p* = 0.165
Postop UPDRS-III	*r* = −0.249	*r* = 0.172	*r* = −0.077	*r* = 0.334
	*p* = 0.170	*p* = 0.340	*p* = 0.677	*p* = 0.057
Postop 3-year LEDD	*r* = −0.007	*r* = 0.142	*r* = 0.208	*r* = 0.262
	*p* = 0.969	*p* = 0.429	*p* = 0.254	*p* = 0.141

Bold values indicate statistically significant associations.

H&Y, Hoehn and Yahr; LEDD, levodopa equivalent daily dose; UPDRS, Unified Parkinson’s Disease Rating Scale.

*p < 0.05 (Spearman rank correlation).

## Discussion

In this study, we found that very-early STN-DBS, performed within 5 years of diagnosis, and early STN-DBS, performed 5 years or more after diagnosis, yielded equivalent long-term motor outcomes as measured by UPDRS-III and H&Y stage at 3-year follow-up. Despite this equivalence in motor outcomes, very-early DBS was associated with a significantly greater reduction in the need for dopaminergic medication, a better early postoperative functional stage, and a weaker association between preoperative symptom duration and subsequent motor outcomes. These findings suggest that differences in surgical timing may not substantially alter long-term motor progression trajectories but may be associated with differences in postoperative medication requirements and early functional outcomes. Importantly, given that both groups underwent surgery relatively early in the disease course compared with historical DBS cohorts, the present comparison may be better interpreted as very-early versus relatively later early-stage DBS rather than early versus advanced-stage intervention. Although the present findings demonstrate associations between earlier surgical timing and postoperative clinical trajectories, the underlying biological mechanisms remain uncertain and cannot be directly inferred from the current observational dataset.

The similar long-term motor outcomes observed across groups reflect the symptomatic nature of DBS: subthalamic nucleus stimulation modulates pathological basal ganglia output without addressing the underlying neurodegenerative process, as shown by similar postoperative courses regardless of when the surgery was performed [11]. The progressive degeneration of non-dopaminergic pathways – including those responsible for axial motor control, cognition, and postural stability – is resistant to both pharmacotherapy and neuromodulation, leading to functional decline regardless of the timing of surgery [11, 19]. The Sydney multicenter study, which prospectively followed a PD cohort for 20 years, demonstrated that dementia, postural instability, and falls are inevitable milestones caused by non-dopaminergic neurodegeneration and are not alleviated by optimizing dopaminergic treatment [19, 20]. This pattern is also reflected in long-term DBS data: a 10-year blinded assessment of STN-DBS reported that motor benefits were largely preserved compared to the preoperative “off” state, but scores for activities of daily living worsened significantly over time – a finding entirely attributable to these stimulation-resistant features [21].

The significant group-by-time interaction for LEDD is a central finding of this study. Both groups converged to comparable postoperative medication levels despite substantially different baseline values, with the early-DBS group showing a larger absolute reduction from its higher starting dose. However, multivariable regression analyses adjusting for age and baseline preoperative LEDD substantially attenuated the between-group difference in LEDD reduction. Baseline preoperative LEDD remained the strongest predictor of postoperative medication reduction across all models, and surgical timing was no longer significantly associated with postoperative LEDD reduction after adjustment. While a residual effect of surgical timing on 3-year LEDD reduction cannot be excluded, the present data do not allow a confident separation between a true timing effect and baseline medication confounding. Accordingly, the magnitude of postoperative LEDD reduction should be interpreted in the context of preoperative medication burden rather than as direct evidence of an independent pharmacological advantage related solely to surgical timing. Merola et al., comparing 203 patients who underwent DBS at different stages of the disease, reported that earlier surgical intervention was associated with earlier control of motor complications and a better course in activities of daily living [7]. In the EARLYSTIM study, patients randomized to DBS within about 7.5 years of disease onset showed greater improvements in quality of life and motor complication burden than those receiving only optimal medical treatment [4]. Hacker et al. [12] 5-year follow-up data confirmed the durability of medication reduction after early DBS; it showed that the LEDD remained consistently lower and motor complications were fewer in the early intervention group. A systematic review and meta-analysis of 8 randomized controlled trials involving 1,189 patients found a significantly greater reduction in LEDD among patients with early-stage PD compared with those with advanced-stage disease; however, there was no difference in UPDRS motor score improvement between the groups [22]. Overall, these findings suggest that earlier intervention may be associated with clinically meaningful pharmacological benefits, although the extent to which these effects are independently attributable to surgical timing remains uncertain. Limousin and Foltynie have noted that reductions in LEDD do not necessarily translate into proportional long-term motor benefit because non-dopaminergic disease progression ultimately becomes the dominant determinant of clinical outcomes regardless of surgical timing [11]. In addition, postoperative medication adjustments in the present cohort were individualized according to routine clinical practice rather than a predefined reduction protocol. Therefore, differences in clinician prescribing strategies, baseline dopaminergic responsiveness, and patient-specific treatment considerations may also have contributed to the observed postoperative LEDD differences between groups. Given the retrospective observational design, these findings should be interpreted as associations rather than definitive causal effects of surgical timing.

One possible explanation for the greater postoperative LEDD reduction observed in the very-early DBS group may relate to intervention before the development of more advanced maladaptive network reorganization within cortico-basal ganglia-thalamocortical circuits [6, 20]. Previous theoretical and experimental models have suggested that prolonged pulsatile dopaminergic stimulation may contribute to abnormal synaptic plasticity and progressive circuit dysfunction over time. Within this conceptual framework, earlier neuromodulatory intervention could hypothetically influence subsequent pharmacological requirements or functional adaptation. As described by Obeso et al., pulsatile dopaminergic stimulation leads to abnormal plasticity in striato-pallidal and subthalamic-pallidal connections over years of pharmacological treatment; performing surgery before these pathological changes become established may enable more effective circuit normalization at lower medication doses [6]. However, the present study was not designed to directly investigate neurophysiological mechanisms, and these interpretations should therefore be considered hypothesis generating rather than definitive mechanistic conclusions. The stronger correlations between preoperative symptom duration and postoperative motor outcomes – which were mostly absent in the very-early DBS group but present in the early DBS group – support this idea of cumulative circuit dysfunction. Janssen et al. found that a longer disease duration at the time of STN-DBS implantation was independently associated with greater functional impairment during follow-up; this is especially true for levodopa-resistant symptoms, indicating that the functional improvements achievable through neuromodulation are limited by the extent of irreversible circuit reorganization that happens before surgery [23].

The statistically significant improvement in the H&Y stage observed in the very-early DBS group during the early postoperative period did not last at the 3-year follow-up. However, the significant group-by-time interaction seen in both the early postoperative and 3-year assessments indicates that both groups experienced a similar decline in function during this period, although the very-early DBS group started from a more favorable baseline. This finding is consistent with the results reported by Hacker et al., who suggested that early DBS may reduce the risk of clinically significant deterioration in early-stage PD, suggesting that a better functional baseline might reduce the impact of ongoing neurodegeneration in the initial years after surgery [8]. It remains uncertain from the current data whether this early benefit will lead to differences in activities of daily living, quality of life, or dependence on care over longer follow-up periods.

Subgroup analyses based on disease phenotype did not show any significant group-time interaction for any motor or functional parameter in either the TD or AR subtypes; this suggests that the similar long-term motor course across timing groups is not influenced by disease subtype [24]. This finding supports the primary analysis and indicates that decisions regarding DBS timing should not be made solely on the motor phenotype.

The relatively higher frequency of PD-plus reclassification observed in the very-early DBS group highlights one of the principal challenges associated with earlier surgical intervention in PD, namely, diagnostic uncertainty during the early stages of parkinsonian syndromes [25]. Although the number of reclassified cases was limited and statistical interpretation should therefore remain cautious, this finding remains clinically important. The MDS clinical diagnostic criteria for PD incorporate a 5-year diagnostic stability concept precisely because atypical clinical features may not yet be fully apparent during earlier disease stages [14]. In our cohort, the majority of reclassified patients initially demonstrated clinical characteristics compatible with PD and underwent surgery following multidisciplinary evaluation; however, longitudinal follow-up later revealed alternative neurodegenerative syndromes including PSP, MSA, and CBS. These observations underscore the importance of careful patient selection, longitudinal reassessment, and cautious interpretation of outcomes when considering DBS at very early disease stages. Importantly, several of the reclassified patients exhibited an initial postoperative symptomatic response despite later development of atypical clinical features. This observation further illustrates the diagnostic complexity of early parkinsonian syndromes and suggests that short-term postoperative improvement alone may not reliably confirm underlying idiopathic PD. These findings reinforce the importance of continued longitudinal reassessment following DBS implantation, particularly in patients undergoing surgery at very early disease stages.

This study has some important limitations. Because it is a retrospective study from a single center, it cannot prove cause and effect. Accordingly, the observed differences between groups should be interpreted as associations identified within a real-world retrospective cohort rather than definitive causal effects of surgical timing. Also, the relationship between surgical timing and outcomes cannot be explained by timing alone since not all influencing factors could be fully controlled. Although groups were matched for age, sex, and preoperative motor severity, residual confounding by unmeasured variables, including cognitive trajectory, non-motor symptom burden, genetic background, and individual dopaminergic responsiveness, cannot be excluded. This residual confounding could have plausibly inflated the apparent LEDD advantage of early DBS if earlier patients had inherently greater baseline dopaminergic responsiveness. In addition, postoperative medication management was individualized rather than protocolized, potentially introducing variability related to clinician prescribing preferences and routine clinical decision-making. The single-center setting limits generalizability to populations with different referral characteristics, varying levels of surgical expertise, or different postoperative programming practices. Disease-subtype analyses are underpowered for detecting differential effects and should be treated as exploratory.

Although longitudinal motor assessments primarily utilized off-medication evaluations, the retrospective real-world design limited complete standardization of medication and stimulation states across all follow-up visits, precluding precise isolation of stimulation-specific effects independent of concurrent pharmacotherapy. Follow-up was limited to 3 years, which may be insufficient to fully capture the long-term trajectory of PD progression and the durability of DBS-related outcomes. Consequently, the present study could not adequately evaluate longer-term clinical milestones including dementia progression, treatment-resistant axial disability, recurrent falls, institutionalization, caregiver dependence, or long-term quality-of-life trajectories. These outcomes may become increasingly relevant during later disease stages and could potentially influence the long-term clinical implications of very-early DBS intervention. An additional important limitation is the absence of standardized quality-of-life and non-motor outcome measures within the retrospective dataset. Instruments such as the Parkinson’s Disease Questionnaire (PDQ-39/PDQ-8) and formal non-motor assessments were not consistently available across the study cohort and therefore could not be reliably incorporated into the analyses. This limitation is particularly relevant in the context of earlier DBS intervention as many of the proposed benefits of earlier surgical treatment relate not only to motor outcomes but also to quality of life, functional independence, and non-motor symptom burden. Consequently, the clinical relevance of the observed differences in medication reduction and functional scores cannot be fully interpreted based solely on motor and medication-related outcomes.

The findings of this study are most relevant for PD patients treated at specialized referral centers with established DBS programs, where multidisciplinary patient selection, standardized surgical techniques, and consistent postoperative programming are conducted by experienced teams. The cohort’s demographic profile, including average age at surgery, gender distribution, and MMSE scores above 24, reflects typical inclusion criteria at academic DBS centers. The findings of this study suggest that, within established patient selection criteria, DBS performed earlier in the disease course may offer a clinically relevant pharmacological advantage without compromising long-term motor efficacy. For patients who meet conventional DBS candidacy criteria, the potential for greater and more sustained medication reduction may represent a meaningful factor in timing discussions, particularly for younger patients with high levodopa burden or early motor fluctuations. Prospective studies with longer follow-up and quality-of-life endpoint analyses are needed to determine whether early DBS also modifies non-motor outcomes or delays functional disability milestones.

In conclusion, both earlier and very earlier STN-DBS were associated with significant and sustained improvement in motor symptoms over the follow-up period, although some functional decline occurred over time consistent with disease progression. The unadjusted between-group difference in LEDD reduction was largely attributable to baseline medication differences rather than surgical timing. Very-early DBS was associated with more favorable early postoperative functional staging, while long-term motor outcomes remained comparable between groups. These findings suggest that, within appropriately selected patients, earlier surgical intervention may represent a feasible therapeutic strategy without compromising long-term motor efficacy. Further prospective studies with longer follow-up, and standardized quality-of-life and non-motor assessments are needed to better define the optimal timing of DBS in PD.

## Statement of Ethics

This study was approved by the Istanbul Medipol University Ethics Committee (Approval No. 852) and conducted in accordance with the Declaration of Helsinki. Written informed consent was obtained from all participants.

## Conflict of Interest Statement

Andres M. Lozano was a member of the journal’s Editorial Board at the time of submission. The remaining authors have no conflicts of interest to declare. B.Ç.A., Ö.K., N.H.Y., M.S.C., Y.E., E.A., Y.S., E.K., and G.K. declare that they have no additional disclosures to report. A.M.L. serves as a consultant for Abbott, Boston Scientific, Insightec, Medtronic, and Functional Neuromodulation and also serves as Scientific Director. T.A.Z. reports relevant proctorship, consultancy, and honorarium relationships with Boston Scientific, NeuroBio, Abbott, and Insightec.

## Funding Sources

The authors received no financial support for this study.

## Author Contributions

B.C.A. and G.K.: drafting of the manuscript, data analysis, and critical revision. Ö.K., N.H.Y., and E.K.: data analysis. M.S.C., Y.E., and E.A.: drafting of the manuscript. Y.S. and A.M.L.: critical revision. T.A.Z.: drafting of the manuscript and critical revision.

## Data Availability

The data that support the findings of this study are not publicly available due to privacy and institutional restrictions but are available from the corresponding author upon reasonable request.
